# Crystal structure of (*E*)-1-(1-hy­droxy­naphthalen-2-yl)-3-(2,3,4-tri­meth­oxy­phen­yl)prop-2-en-1-one

**DOI:** 10.1107/S2056989015013870

**Published:** 2015-07-29

**Authors:** J. Srividya, D. Reuben Jonathan, B. K. Revathi, G. Anbalagan

**Affiliations:** a PG and Research Department of Physics, Queen Mary’s College, Chennai 600 004, India; b Department of Chemistry, Madras Christian College, Chennai-59, India; c PG and Research Department of Physics, Presidency College, University of Madras, Chennai 600 005, India

**Keywords:** crystal structure, chalcones, hy­droxy­naphthalene, O—H⋯O hydrogen bond, *S*(6) ring motif, π–π slipped parallel inter­action

## Abstract

The title compound, C_22_H_20_O_5_, is composed of a hy­droxy­naphthyl ring and a tri­meth­oxy­phenyl ring [the planes of which are inclined to one another by 21.61 (10)°] bridged by an unsaturated prop-2-en-1-one group. The mean plane of the prop-2-en-1-one group [–C(=O)—C=C–] is inclined to that of the naphthyl system and benzene rings by 3.77 (14) and 18.01 (16)°, respectively. There is an intra­molecular O—H⋯O hydrogen bond present forming an *S*(6) ring motif. In the crystal, inversion-related mol­ecules are linked by a slipped-parallel π–π inter­action [inter­centroid distance = 3.8942 (13) Å, inter­planar distance = 3.478 (9) Å and slippage = 1.751 Å], and stack along the [101] direction. There are no other significant inter­molecular inter­actions present.

## Related literature   

For natural sources of chalcones and flavonoids, see: Anderson & Markham (2006 ▸); Yadav *et al.* (2011 ▸). For their biological activities, see: Lin *et al.* (2002 ▸); Dhar (1981 ▸); Mukherjee *et al.* (2001 ▸); Bhat *et al.* (2005 ▸); Go *et al.* (2005 ▸); Sashidhara *et al.* (2011 ▸). For the synthesis by Claisen–Schmidt reaction, see: Shettigar *et al.* (2006 ▸); Ezhilarasi *et al.* (2015 ▸). For related structures, see: Wu *et al.* (2005 ▸); Lu *et al.* (2006 ▸); Harrison *et al.* (2007 ▸); Ezhilarasi *et al.* (2015 ▸).
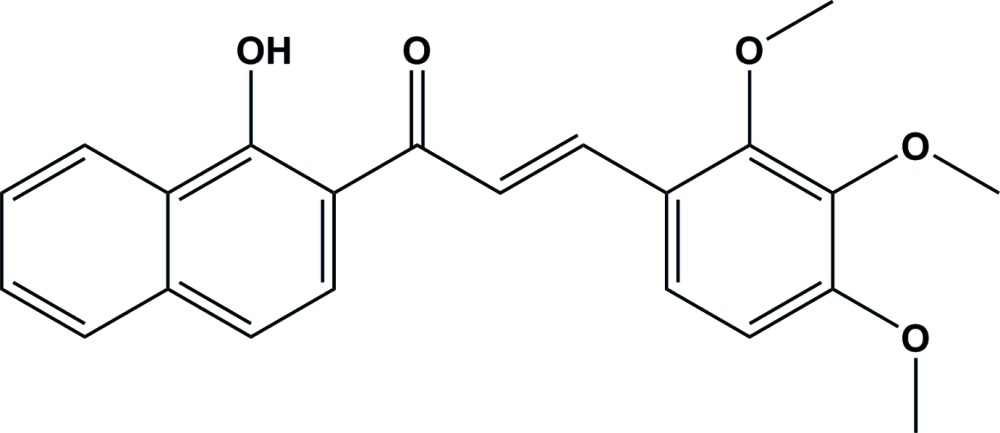


## Experimental   

### Crystal data  

C_22_H_20_O_5_
*M* *_r_* = 364.38
Monoclinic, 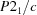

*a* = 8.4523 (8) Å
*b* = 14.0414 (12) Å
*c* = 15.1672 (11) Å
β = 94.623 (3)°
*V* = 1794.2 (3) Å^3^
*Z* = 4
Mo *K*α radiation
μ = 0.10 mm^−1^
*T* = 293 K
0.35 × 0.30 × 0.25 mm

### Data collection  

Bruker Kappa APEXII CCD diffractometer
Absorption correction: multi-scan (*SADABS*; Bruker, 2004 ▸) *T*_min_ = 0.967, *T*_max_ = 0.977
19676 measured reflections
3151 independent reflections
2143 reflections with *I* > 2σ(*I*)
*R*_int_ = 0.033

### Refinement  

*R*[*F* ^2^ > 2σ(*F* ^2^)] = 0.044
*wR*(*F* ^2^) = 0.144
*S* = 1.08
3151 reflections
245 parameters
H-atom parameters constrained
Δρ_max_ = 0.19 e Å^−3^
Δρ_min_ = −0.18 e Å^−3^

### 

Data collection: *APEX2* (Bruker, 2004 ▸); cell refinement: *APEX2* and *SAINT* (Bruker, 2004 ▸); data reduction: *SAINT* and *XPREP* (Bruker, 2004 ▸); program(s) used to solve structure: *SIR92* (Altomare *et al.*, 1994 ▸); program(s) used to refine structure: *SHELXL2014* (Sheldrick, 2015 ▸); molecular graphics: *ORTEP-3 for Windows* (Farrugia, 2012 ▸) and *Mercury* (Macrae *et al.*, 2008 ▸); software used to prepare material for publication: *SHELXL2014* and *PLATON* (Spek, 2009 ▸).

## Supplementary Material

Crystal structure: contains datablock(s) I, New_Global_Publ_Block. DOI: 10.1107/S2056989015013870/su5174sup1.cif

Structure factors: contains datablock(s) I. DOI: 10.1107/S2056989015013870/su5174Isup2.hkl

Supporting information file. DOI: 10.1107/S2056989015013870/su5174Isup3.cml

**Fig. 1.**
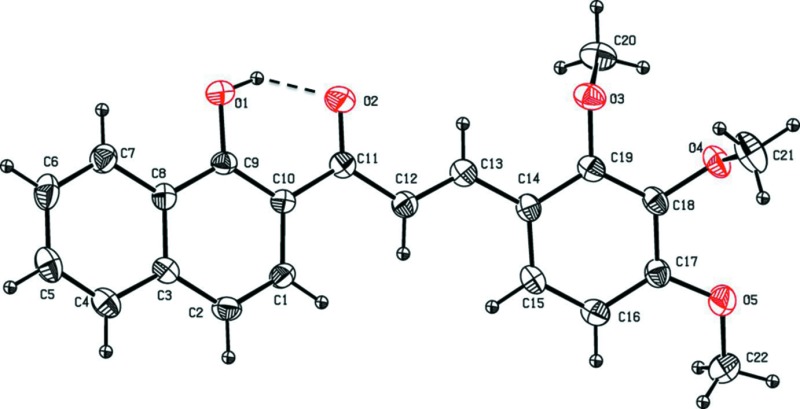
The molecular structure of the title compound, showing the atom labelling. Displacement ellipsoids are drawn at the 30% probability level. The intramolecular O—H···O hydrogen bond is shown as a dashed line (see Table 1).

**Fig. 2.**
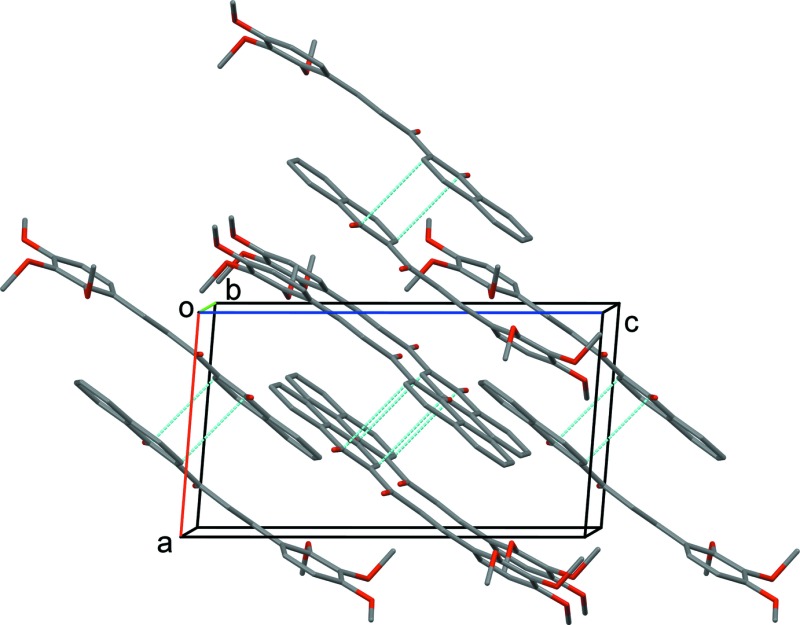
The crystal packing of the title compound, viewed along the b axis. The dashed lines indicate the π–π interactions involving inversion-related molecules. H atoms have been omitted for clarity.

. DOI: 10.1107/S2056989015013870/su5174fig1.tif
The mol­ecular structure of the title compound, showing the atom labelling. Displacement ellipsoids are drawn at the 30% probability level. The intra­molecular O—H⋯O hydrogen bond is shown as a dashed line (see Table 1).

b . DOI: 10.1107/S2056989015013870/su5174fig2.tif
The crystal packing of the title compound, viewed along the *b* axis. The dashed lines indicate the π–π inter­actions involving inversion-related mol­ecules. H atoms have been omitted for clarity.

CCDC reference: 1414195

Additional supporting information:  crystallographic information; 3D view; checkCIF report

## Figures and Tables

**Table 1:** Hydrogen-bond geometry (, )

*D*H*A*	*D*H	H*A*	*D* *A*	*D*H*A*
O1H7O2	0.82	1.77	2.500(2)	147

## supplementary crystallographic information

### S1. Chemical context

Chalcones have characteristic 1,3-di­aryl-2-propen-1-one skeleton and occur naturally in roots, rhizomes, heartwood, leaves, petal pigments and seeds of many kinds of flora (Anderson & Markham, 2006; Yadav *et al.*, 2011). Chalcones and chalconoid derivatives originate from the open chain flavonoid family form a wide spectrum of bioactive compounds exhibiting cytoprotective and immuno-modulatory functions (Anderson & Markham, 2006; Lin *et al.*, 2002). The unsaturated ketone moiety, the conjugated double bonds and the radical quenching property of the phenolic group, presents versatile anti-inflammatory (Sashidhara *et al.*, 2011), anti­bacterial, anti-oxidant (Mukherjee *et al.*, 2001), anti­fungal (Go *et al.*, 2005) and anti­cancerous properties (Dhar, 1981; Bhat *et al.*, 2005). The type of substitutuent group and their pattern are linked closely to their pharmacological applications.

### S2. Structural commentary

The molecular structure of the title compound is illustrated in Fig. 1. The bond lengths and angles are similar to those reported for the above-mentioned compounds. Atoms O3, O4 and O5 of the meth­oxy groups deviate from the benzene ring by -0.032 (2), 0.026 (2) and -0.012 (2) Å, respectively. The dihedral angle between the planes of the naphthyl system and benzene ring is 21.61 (10)°, and those between the mean plane of the prop-2-en-1-one group [–C11(═O2)—C12═C13–] and those of the naphthyl system and benzene ring are 3.77 (14) and 18.01 (16)°, respectively. The C22—O5—C17—C18 , C20—O3—C19—C18 and C21—O4—C18—C19 torsion angles [164.3 (2), -73.7 (3) and -87.1 (3)°, respectively] indicate +ap, -sc and -sc orientations of the meth­oxy groups with respect to the benzene ring.

### S3. Supra­molecular features

In the crystal (Fig. 2), the meth­oxy groups substituted in the 3- and 4-positions of the benzene ring allows stacking along [101], rather than close packing of the molecules. Inversion-related molecules are linked by a slipped parallel π–π inter­action [*Cg*1···*Cg*1^i^ = 3.8942 (13) Å, inter­planar distance = 3.478 (9) Å and slippage = 1.751 Å; *Cg*1 is the centroid of C1–C3/C8–C10 ring ; symmetry code: (i) -*x*+1, -*y*+1, -*z*]. There are no other significant inter­molecular inter­actions present.

In comparison to the crystal structure reported for (*E*)-1-(2-naphthyl)-3-(3,4,5-tri­meth­oxy­phenyl)­prop-2-en-1-one (Lu *et al.*, 2006), the presence of an intra­molecular O—H···O hydrogen bond (Table 1) between the hy­droxy group and the propenone O atom in the title compound gives steric planar stability to the molecule in the *E* conformation and restricts the bending of the molecule.

### S4. Database survey

\ A series of related structures have been reported, *viz.* (*E*)-1-(2-hy­droxy­phenyl)-3-(3,4,5-tri­meth­oxy­phenyl)­prop-2-en-1-one (Wu *et al.*, 2005), (*E*)-1-(2-naphthyl)-3-(3,4,5-tri­meth­oxy­phenyl)­prop-2-en-1-one (Lu *et al.*, 2006), 1-(4-hy­droxy­phenyl)-3-(3,4,5-tri­meth­oxy­phenyl)­prop-2-en-1-one (Harrison *et al.*, 2007) and (*E*)-3-(3,4-di­meth­oxy­phenyl)-1-(1-hy­droxy­naphthalen-2-yl)prop-2-en-1-one (Ezhilarasi *et al.*, 2015). The synthesis and crystal structure of a new similar chalcone analogue, namely (*E*)-1-(1-hy­droxy­naphthalen-2-yl)-3-(2,3,4-tri­meth­oxy­phenyl)­prop-2-en-\ 1-one, are reported here.

### S5. Synthesis and crystallization

The title compound was synthesized by Claisen–Schmidt reaction (Shettigar *et al.*, 2006; Ezhilarasi *et al.*, 2015). About 2 mmol of 1-(1-hy­droxy-2-naphthyl)­ethanone was added to 2,3,4-tri­meth­oxy­benzaldehyde (2 mmol) in a 250ml round-bottomed flask and the mixture was dissolved in 100 ml of absolute ethanol through constant stirring. A 10% sodium hydroxide solution (20 ml) was then added to this homogeneous mixture with continuous stirring for 24 h, which was initially pale-yellow but turned orange–red. The progress of the reaction was monitored by thin-layer chromatography (TLC) and upon completion of the reaction, the final mixture was quenched by pouring it into an ice-cold 10% solution of HCl (pH = 3) to precipitate the crude product. The orange precipitate was filtered off, washed with distilled water and dried at room temperature. The crude product after extraction with ethyl acetate was recrystallized with chloro­form and allowed to evaporate slowly in a constant-temperature bath to give orange good-quality block-like crystals after 10 d (yield 79.8%; m.p. 394–395 K).

### S6. Refinement

Crystal data, data collection and structure refinement details are summarized in Table 2. All H atoms were positioned geometrically and refined as riding atoms: O—H = 0.82 Å and C—H = 0.93–0.98 Å, with *U*_iso_(H) = 1.5*U*_eq_(O,C) for hy­droxy and methyl H atoms, and 1.2*U*_eq_(C) for the other H atoms.

### Crystal data

**Table:**

C_22_H_20_O_5_	*F*(000) = 768
*M**_r_* = 364.38	*D*_x_ = 1.349 Mg m^−^^3^
Monoclinic, *P*2_1_/*c*	Melting point: 394(2) K
Hall symbol: -P 2ybc	Mo *K*α radiation, λ = 0.71073 Å
*a* = 8.4523 (8) Å	θ = 2.0–25.0°
*b* = 14.0414 (12) Å	µ = 0.10 mm^−^^1^
*c* = 15.1672 (11) Å	*T* = 293 K
β = 94.623 (3)°	Block, orange
*V* = 1794.2 (3) Å^3^	0.35 × 0.30 × 0.25 mm
*Z* = 4	

### Data collection

**Table:**

Bruker Kappa APEXII CCD diffractometer	3151 independent reflections
Radiation source: fine-focus sealed tube	2143 reflections with *I* > 2σ(*I*)
Graphite monochromator	*R*_int_ = 0.033
ω and φ scan	θ_max_ = 25.0°, θ_min_ = 2.0°
Absorption correction: multi-scan (*SADABS*; Bruker, 2004)	*h* = −10→10
*T*_min_ = 0.967, *T*_max_ = 0.977	*k* = −16→16
19676 measured reflections	*l* = −17→18

### Refinement

**Table:**

Refinement on *F*^2^	Secondary atom site location: difference Fourier map
Least-squares matrix: full	Hydrogen site location: inferred from neighbouring sites
*R*[*F*^2^ > 2σ(*F*^2^)] = 0.044	H-atom parameters constrained
*wR*(*F*^2^) = 0.144	*w* = 1/[σ^2^(*F*_o_^2^) + (0.0626*P*)^2^ + 0.7956*P*] where *P* = (*F*_o_^2^ + 2*F*_c_^2^)/3
*S* = 1.08	(Δ/σ)_max_ < 0.001
3151 reflections	Δρ_max_ = 0.19 e Å^−^^3^
245 parameters	Δρ_min_ = −0.18 e Å^−^^3^
0 restraints	Extinction correction: *SHELXL2014* (Sheldrick, 2015), Fc^*^=kFc[1+0.001xFc^2^λ^3^/sin(2θ)]^-1/4^
Primary atom site location: structure-invariant direct methods	Extinction coefficient: 0.0031 (9)

### Special details

**Table:**

Geometry. All e.s.d.'s (except the e.s.d. in the dihedral angle between two l.s. planes) are estimated using the full covariance matrix. The cell e.s.d.'s are taken into account individually in the estimation of e.s.d.'s in distances, angles and torsion angles; correlations between e.s.d.'s in cell parameters are only used when they are defined by crystal symmetry. An approximate (isotropic) treatment of cell e.s.d.'s is used for estimating e.s.d.'s involving l.s. planes.
Refinement. Refinement of *F*^2^ against ALL reflections. The weighted *R*-factor *wR* and goodness of fit *S* are based on *F*^2^, conventional *R*-factors *R* are based on *F*, with *F* set to zero for negative *F*^2^. The threshold expression of *F*^2^ > σ(*F*^2^) is used only for calculating *R*-factors(gt) *etc*. and is not relevant to the choice of reflections for refinement. *R*-factors based on *F*^2^ are statistically about twice as large as those based on *F*, and *R*- factors based on ALL data will be even larger.

### Fractional atomic coordinates and isotropic or equivalent isotropic displacement parameters (Å^2^)

**Table:**

	*x*	*y*	*z*	*U*_iso_*/*U*_eq_	
O1	0.3943 (2)	0.35902 (11)	0.13288 (11)	0.0535 (5)	
H7	0.3368	0.3315	0.0949	0.080*	
O2	0.2007 (2)	0.34197 (12)	0.00026 (12)	0.0590 (5)	
C10	0.3050 (2)	0.49116 (15)	0.04358 (14)	0.0363 (5)	
O3	−0.05999 (19)	0.28866 (11)	−0.28956 (12)	0.0522 (5)	
C8	0.4894 (2)	0.51062 (16)	0.17556 (14)	0.0384 (5)	
O4	−0.2147 (2)	0.35002 (12)	−0.44607 (11)	0.0556 (5)	
C9	0.3937 (2)	0.45260 (15)	0.11569 (14)	0.0374 (5)	
C12	0.1103 (3)	0.46876 (17)	−0.09075 (15)	0.0441 (6)	
H8	0.1033	0.5346	−0.0966	0.053*	
C18	−0.1771 (3)	0.41365 (16)	−0.37851 (15)	0.0429 (6)	
C3	0.4953 (3)	0.60934 (16)	0.15994 (15)	0.0412 (6)	
C14	−0.0573 (3)	0.44733 (16)	−0.23078 (15)	0.0408 (6)	
C11	0.2047 (3)	0.42933 (17)	−0.01508 (15)	0.0410 (6)	
C13	0.0336 (3)	0.41439 (17)	−0.15184 (15)	0.0440 (6)	
H9	0.0389	0.3489	−0.1430	0.053*	
C19	−0.1002 (3)	0.38331 (16)	−0.29973 (15)	0.0422 (6)	
C2	0.4064 (3)	0.64762 (16)	0.08524 (16)	0.0475 (6)	
H2	0.4108	0.7126	0.0740	0.057*	
C15	−0.1021 (3)	0.54137 (17)	−0.24414 (15)	0.0447 (6)	
H10	−0.0758	0.5853	−0.1995	0.054*	
C1	0.3154 (3)	0.59125 (16)	0.03021 (15)	0.0439 (6)	
H1	0.2575	0.6185	−0.0181	0.053*	
C16	−0.1836 (3)	0.57236 (17)	−0.32046 (16)	0.0483 (6)	
H11	−0.2129	0.6360	−0.3265	0.058*	
C4	0.5891 (3)	0.66571 (19)	0.22005 (17)	0.0523 (7)	
H3	0.5934	0.7311	0.2111	0.063*	
O5	−0.3020 (2)	0.53170 (13)	−0.46730 (11)	0.0612 (5)	
C17	−0.2225 (3)	0.50847 (17)	−0.38884 (15)	0.0452 (6)	
C6	0.6668 (3)	0.5294 (2)	0.30668 (17)	0.0611 (7)	
H5	0.7240	0.5033	0.3558	0.073*	
C7	0.5762 (3)	0.47234 (19)	0.25003 (16)	0.0510 (6)	
H6	0.5718	0.4072	0.2609	0.061*	
C5	0.6735 (3)	0.6270 (2)	0.29078 (18)	0.0588 (7)	
H4	0.7363	0.6657	0.3290	0.071*	
C22	−0.3846 (3)	0.6192 (2)	−0.47248 (19)	0.0649 (8)	
H13	−0.4353	0.6271	−0.5310	0.097*	
H14	−0.3111	0.6705	−0.4597	0.097*	
H12	−0.4633	0.6196	−0.4303	0.097*	
C20	−0.1934 (3)	0.22848 (19)	−0.2780 (2)	0.0645 (8)	
H19	−0.1584	0.1637	−0.2712	0.097*	
H20	−0.2684	0.2335	−0.3288	0.097*	
H18	−0.2430	0.2479	−0.2261	0.097*	
C21	−0.0907 (4)	0.3367 (2)	−0.5025 (2)	0.0822 (10)	
H16	−0.1239	0.2918	−0.5480	0.123*	
H15	0.0018	0.3129	−0.4687	0.123*	
H17	−0.0661	0.3964	−0.5290	0.123*	

### Atomic displacement parameters (Å^2^)

**Table:**

	*U*^11^	*U*^22^	*U*^33^	*U*^12^	*U*^13^	*U*^23^
O1	0.0681 (12)	0.0373 (9)	0.0539 (10)	0.0006 (8)	−0.0029 (9)	0.0036 (8)
O2	0.0711 (13)	0.0404 (11)	0.0636 (12)	−0.0109 (9)	−0.0066 (9)	0.0000 (8)
C10	0.0351 (12)	0.0352 (12)	0.0392 (12)	0.0002 (9)	0.0064 (10)	0.0003 (9)
O3	0.0430 (10)	0.0425 (10)	0.0702 (12)	0.0030 (8)	−0.0010 (8)	−0.0069 (8)
C8	0.0321 (12)	0.0452 (14)	0.0384 (12)	0.0042 (10)	0.0059 (9)	−0.0026 (10)
O4	0.0494 (10)	0.0588 (11)	0.0570 (11)	−0.0008 (8)	−0.0054 (8)	−0.0205 (9)
C9	0.0405 (12)	0.0315 (12)	0.0413 (13)	0.0040 (9)	0.0100 (10)	0.0012 (10)
C12	0.0442 (13)	0.0434 (14)	0.0448 (14)	−0.0025 (11)	0.0036 (11)	−0.0007 (11)
C18	0.0334 (12)	0.0464 (14)	0.0483 (14)	−0.0037 (10)	−0.0010 (10)	−0.0130 (11)
C3	0.0366 (12)	0.0419 (13)	0.0458 (14)	−0.0017 (10)	0.0082 (10)	−0.0057 (11)
C14	0.0333 (12)	0.0466 (14)	0.0426 (13)	−0.0035 (10)	0.0037 (10)	−0.0055 (11)
C11	0.0388 (13)	0.0424 (14)	0.0426 (13)	−0.0027 (10)	0.0083 (10)	−0.0004 (10)
C13	0.0399 (13)	0.0453 (14)	0.0471 (14)	−0.0021 (10)	0.0052 (11)	−0.0023 (11)
C19	0.0328 (12)	0.0415 (13)	0.0522 (15)	−0.0016 (10)	0.0032 (10)	−0.0056 (11)
C2	0.0502 (14)	0.0339 (13)	0.0576 (16)	−0.0030 (11)	0.0006 (12)	0.0023 (11)
C15	0.0434 (13)	0.0462 (14)	0.0446 (14)	−0.0043 (11)	0.0039 (11)	−0.0127 (11)
C1	0.0460 (14)	0.0411 (14)	0.0436 (13)	0.0008 (11)	−0.0021 (11)	0.0057 (11)
C16	0.0473 (14)	0.0426 (14)	0.0549 (16)	0.0004 (11)	0.0042 (12)	−0.0056 (12)
C4	0.0461 (14)	0.0523 (16)	0.0586 (16)	−0.0045 (12)	0.0044 (12)	−0.0109 (12)
O5	0.0691 (12)	0.0600 (12)	0.0521 (11)	0.0124 (9)	−0.0095 (9)	−0.0049 (9)
C17	0.0394 (13)	0.0512 (15)	0.0445 (14)	0.0007 (11)	0.0009 (10)	−0.0041 (11)
C6	0.0545 (16)	0.082 (2)	0.0450 (15)	0.0108 (15)	−0.0073 (12)	−0.0051 (14)
C7	0.0519 (15)	0.0547 (16)	0.0463 (14)	0.0083 (12)	0.0035 (12)	0.0014 (12)
C5	0.0469 (15)	0.073 (2)	0.0553 (17)	−0.0034 (14)	−0.0007 (13)	−0.0192 (14)
C22	0.0716 (19)	0.0591 (17)	0.0624 (18)	0.0114 (15)	−0.0036 (14)	0.0067 (14)
C20	0.0590 (17)	0.0483 (16)	0.087 (2)	−0.0054 (13)	0.0098 (15)	−0.0017 (14)
C21	0.079 (2)	0.096 (2)	0.073 (2)	−0.0078 (18)	0.0174 (17)	−0.0383 (18)

### Geometric parameters (Å, º)

**Table:**

O1—C9	1.340 (3)	C2—H2	0.9300
O1—H7	0.8200	C15—C16	1.369 (3)
O2—C11	1.250 (3)	C15—H10	0.9300
C10—C9	1.386 (3)	C1—H1	0.9300
C10—C1	1.424 (3)	C16—C17	1.390 (3)
C10—C11	1.464 (3)	C16—H11	0.9300
O3—C19	1.377 (3)	C4—C5	1.354 (4)
O3—C20	1.431 (3)	C4—H3	0.9300
C8—C7	1.403 (3)	O5—C17	1.359 (3)
C8—C3	1.408 (3)	O5—C22	1.413 (3)
C8—C9	1.423 (3)	C6—C7	1.365 (4)
O4—C18	1.377 (3)	C6—C5	1.392 (4)
O4—C21	1.418 (3)	C6—H5	0.9300
C12—C13	1.328 (3)	C7—H6	0.9300
C12—C11	1.454 (3)	C5—H4	0.9300
C12—H8	0.9300	C22—H13	0.9600
C18—C19	1.381 (3)	C22—H14	0.9600
C18—C17	1.391 (3)	C22—H12	0.9600
C3—C4	1.404 (3)	C20—H19	0.9600
C3—C2	1.414 (3)	C20—H20	0.9600
C14—C15	1.384 (3)	C20—H18	0.9600
C14—C19	1.404 (3)	C21—H16	0.9600
C14—C13	1.445 (3)	C21—H15	0.9600
C13—H9	0.9300	C21—H17	0.9600
C2—C1	1.346 (3)		
			
C9—O1—H7	109.5	C2—C1—H1	119.0
C9—C10—C1	117.5 (2)	C10—C1—H1	119.0
C9—C10—C11	119.9 (2)	C15—C16—C17	119.9 (2)
C1—C10—C11	122.6 (2)	C15—C16—H11	120.1
C19—O3—C20	113.18 (18)	C17—C16—H11	120.1
C7—C8—C3	119.3 (2)	C5—C4—C3	121.5 (3)
C7—C8—C9	121.8 (2)	C5—C4—H3	119.3
C3—C8—C9	118.8 (2)	C3—C4—H3	119.3
C18—O4—C21	113.39 (19)	C17—O5—C22	117.7 (2)
O1—C9—C10	122.0 (2)	O5—C17—C16	124.7 (2)
O1—C9—C8	116.40 (19)	O5—C17—C18	116.2 (2)
C10—C9—C8	121.6 (2)	C16—C17—C18	119.2 (2)
C13—C12—C11	122.5 (2)	C7—C6—C5	119.8 (2)
C13—C12—H8	118.7	C7—C6—H5	120.1
C11—C12—H8	118.7	C5—C6—H5	120.1
O4—C18—C19	120.5 (2)	C6—C7—C8	120.8 (3)
O4—C18—C17	119.5 (2)	C6—C7—H6	119.6
C19—C18—C17	119.9 (2)	C8—C7—H6	119.6
C4—C3—C8	118.1 (2)	C4—C5—C6	120.4 (2)
C4—C3—C2	122.8 (2)	C4—C5—H4	119.8
C8—C3—C2	119.0 (2)	C6—C5—H4	119.8
C15—C14—C19	116.8 (2)	O5—C22—H13	109.5
C15—C14—C13	123.2 (2)	O5—C22—H14	109.5
C19—C14—C13	120.0 (2)	H13—C22—H14	109.5
O2—C11—C12	120.0 (2)	O5—C22—H12	109.5
O2—C11—C10	119.5 (2)	H13—C22—H12	109.5
C12—C11—C10	120.5 (2)	H14—C22—H12	109.5
C12—C13—C14	126.2 (2)	O3—C20—H19	109.5
C12—C13—H9	116.9	O3—C20—H20	109.5
C14—C13—H9	116.9	H19—C20—H20	109.5
O3—C19—C18	119.3 (2)	O3—C20—H18	109.5
O3—C19—C14	119.2 (2)	H19—C20—H18	109.5
C18—C19—C14	121.5 (2)	H20—C20—H18	109.5
C1—C2—C3	120.9 (2)	O4—C21—H16	109.5
C1—C2—H2	119.6	O4—C21—H15	109.5
C3—C2—H2	119.6	H16—C21—H15	109.5
C16—C15—C14	122.7 (2)	O4—C21—H17	109.5
C16—C15—H10	118.7	H16—C21—H17	109.5
C14—C15—H10	118.7	H15—C21—H17	109.5
C2—C1—C10	122.1 (2)		
			
C1—C10—C9—O1	−178.69 (19)	C17—C18—C19—C14	−4.0 (3)
C11—C10—C9—O1	1.6 (3)	C15—C14—C19—O3	−179.34 (19)
C1—C10—C9—C8	1.3 (3)	C13—C14—C19—O3	2.5 (3)
C11—C10—C9—C8	−178.34 (19)	C15—C14—C19—C18	2.8 (3)
C7—C8—C9—O1	−1.8 (3)	C13—C14—C19—C18	−175.3 (2)
C3—C8—C9—O1	179.03 (18)	C4—C3—C2—C1	−178.7 (2)
C7—C8—C9—C10	178.2 (2)	C8—C3—C2—C1	0.9 (3)
C3—C8—C9—C10	−1.0 (3)	C19—C14—C15—C16	−0.4 (3)
C21—O4—C18—C19	−87.1 (3)	C13—C14—C15—C16	177.7 (2)
C21—O4—C18—C17	95.3 (3)	C3—C2—C1—C10	−0.6 (4)
C7—C8—C3—C4	0.2 (3)	C9—C10—C1—C2	−0.6 (3)
C9—C8—C3—C4	179.44 (19)	C11—C10—C1—C2	179.1 (2)
C7—C8—C3—C2	−179.4 (2)	C14—C15—C16—C17	−0.9 (4)
C9—C8—C3—C2	−0.2 (3)	C8—C3—C4—C5	0.7 (3)
C13—C12—C11—O2	−8.7 (3)	C2—C3—C4—C5	−179.7 (2)
C13—C12—C11—C10	171.3 (2)	C22—O5—C17—C16	−16.3 (4)
C9—C10—C11—O2	−0.3 (3)	C22—O5—C17—C18	164.3 (2)
C1—C10—C11—O2	−180.0 (2)	C15—C16—C17—O5	−179.6 (2)
C9—C10—C11—C12	179.7 (2)	C15—C16—C17—C18	−0.2 (4)
C1—C10—C11—C12	0.1 (3)	O4—C18—C17—O5	−0.3 (3)
C11—C12—C13—C14	−177.3 (2)	C19—C18—C17—O5	−177.9 (2)
C15—C14—C13—C12	−13.3 (4)	O4—C18—C17—C16	−179.8 (2)
C19—C14—C13—C12	164.7 (2)	C19—C18—C17—C16	2.6 (3)
C20—O3—C19—C18	−73.7 (3)	C5—C6—C7—C8	0.1 (4)
C20—O3—C19—C14	108.4 (2)	C3—C8—C7—C6	−0.6 (3)
O4—C18—C19—O3	0.6 (3)	C9—C8—C7—C6	−179.8 (2)
C17—C18—C19—O3	178.2 (2)	C3—C4—C5—C6	−1.2 (4)
O4—C18—C19—C14	178.4 (2)	C7—C6—C5—C4	0.8 (4)

### Hydrogen-bond geometry (Å, º)

**Table:**

*D*—H···*A*	*D*—H	H···*A*	*D*···*A*	*D*—H···*A*
O1—H7···O2	0.82	1.77	2.500 (2)	147

## References

[bb1] Altomare, A., Cascarano, G., Giacovazzo, C., Guagliardi, A., Burla, M. C., Polidori, G. & Camalli, M. (1994). *J. Appl. Cryst.* **27**, 435.

[bb2] Anderson, O. M. & Markham, K. R. (2006). In *Flavonoids Chemistry, Biochemistry and Applications*. New York: Taylor and Francis.

[bb3] Bhat, B. A., Dhar, K. L., Puri, S. C., Saxena, A. K., Shanmugavel, M. & Qazi, G. N. (2005). *Bioorg. Med. Chem. Lett.* **15**, 3177–3180. 10.1016/j.bmcl.2005.03.121 15893928

[bb4] Bruker (2004). *APEX2*, *SAINT*, *XPREP* and *SADABS*. Bruker AXS Inc., Madison, Wisconsin, USA.

[bb5] Dhar, D. N. (1981). In *The Chemistry of Chalcones and Related Compounds*. New York: John Wiley.

[bb6] Ezhilarasi, K. S., Reuben Jonathan, D., Vasanthi, R., Revathi, B. K. & Usha, G. (2015). *Acta Cryst.* E**71**, o371–o372. 10.1107/S2056989015008087 PMC4420054 25995955

[bb7] Farrugia, L. J. (2012). *J. Appl. Cryst.* **45**, 849–854.

[bb8] Go, M. L., Wu, X. & Liu, X. L. (2005). *Curr. Med. Chem.* **12**, 483–499.

[bb9] Harrison, W. T. A., Kumari, V., Ravindra, H. J. & Dharmaprakash, S. M. (2007). *Acta Cryst.* E**63**, o2928. 10.1107/S0108270107021774 17609553

[bb10] Lin, L. C., Kuo, Y. C. & Chou, C. J. (2002). *J. Nat. Prod.* **65**, 505–508. 10.1021/np010414l 11975489

[bb11] Lu, Z.-K., Huang, P.-M. & Yu, J.-F. (2006). *Acta Cryst.* E**62**, o5753–o5754.

[bb12] Macrae, C. F., Bruno, I. J., Chisholm, J. A., Edgington, P. R., McCabe, P., Pidcock, E., Rodriguez-Monge, L., Taylor, R., van de Streek, J. & Wood, P. A. (2008). *J. Appl. Cryst.* **41**, 466–470.

[bb13] Mukherjee, S., Kumar, V., Prasad, A. K., Raj, H. G., Bracke, M. E., Olsen, C. E., Jain, S. C. & Parmar, V. S. (2001). *Bioorg. Med. Chem.* **9**, 337–345. 10.1016/s0968-0896(00)00249-2 11249126

[bb14] Sashidhara, K. V., Kumar, M., Modukuri, R. M., Sonkar, R., Bhatia, G., Khanna, A. K., Rai, S. V. & Shukla, R. (2011). *Bioorg. Med. Chem. Lett.* **21**, 4480–4484. 10.1016/j.bmcl.2011.06.002 21723119

[bb15] Sheldrick, G. M. (2015). *Acta Cryst.* C**71**, 3–8.

[bb16] Shettigar, V., Patil, P. S., Naveen, S., Dharmaprakash, S. M., Sridhar, M. A. & Shashidhara Prasad, J. (2006). *J. Cryst. Growth*, **295**, 44–49.

[bb17] Spek, A. L. (2009). *Acta Cryst.* D**65**, 148–155. 10.1107/S090744490804362X PMC2631630 19171970

[bb18] Wu, H., Xu, Z. & Liang, Y.-M. (2005). *Acta Cryst.* E**61**, o1434–o1435.

[bb19] Yadav, V. R., Prasad, S., Sung, B. & Aggarwal, B. B. (2011). *Int. Immunopharmacol.* **11**, 295–309. 10.1016/j.intimp.2010.12.006 PMC3058688 21184860

