# Supplementary figures and images for: Crystal structure of (E)-1-(1-hy­droxy­naphthalen-2-yl)-3-(2,3,4-tri­meth­oxy­phen­yl)prop-2-en-1-one
Source: Acta Crystallogr E Crystallogr Commun. 2015 Jul 29;71(Pt 8):o610–1. doi: 10.1107/S2056989015013870 (PMC4571423; doi:10.1107/S2056989015013870)

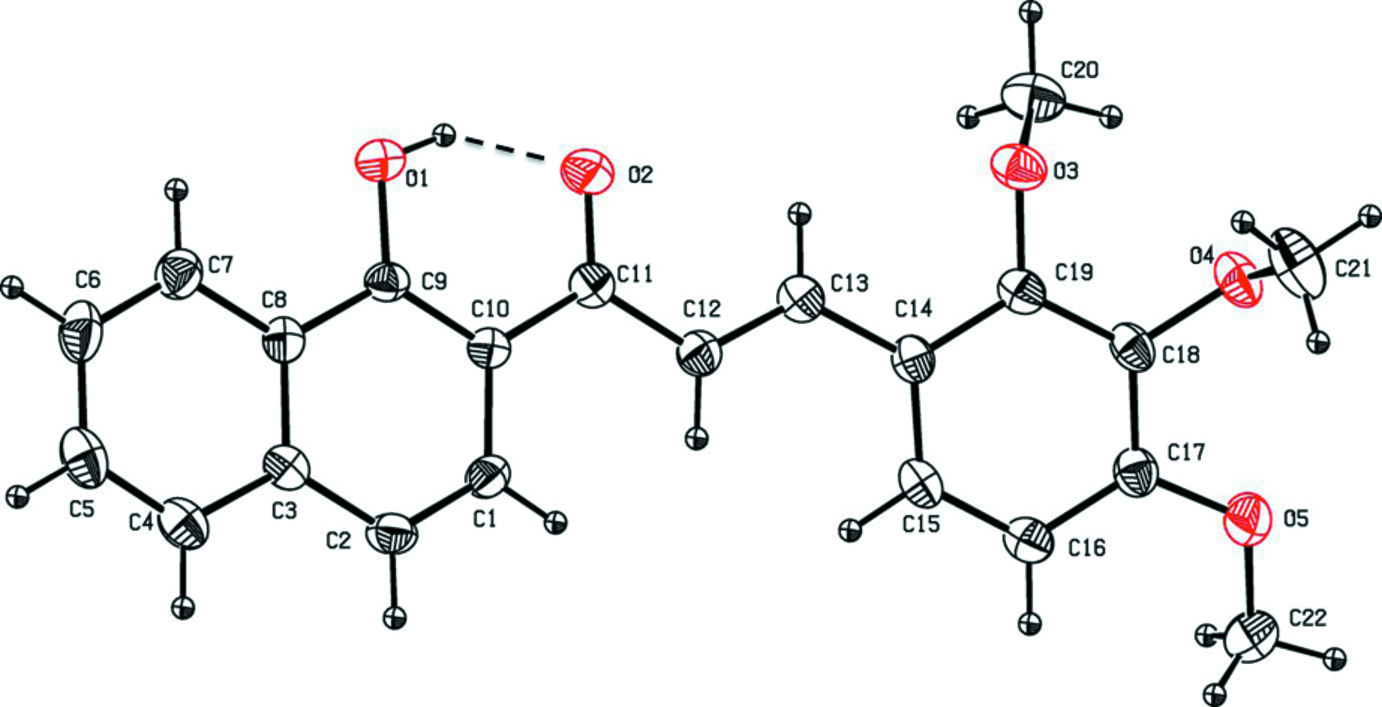

Supplement: Supplementary file 4 [file e-71-0o610-fig1.tif]

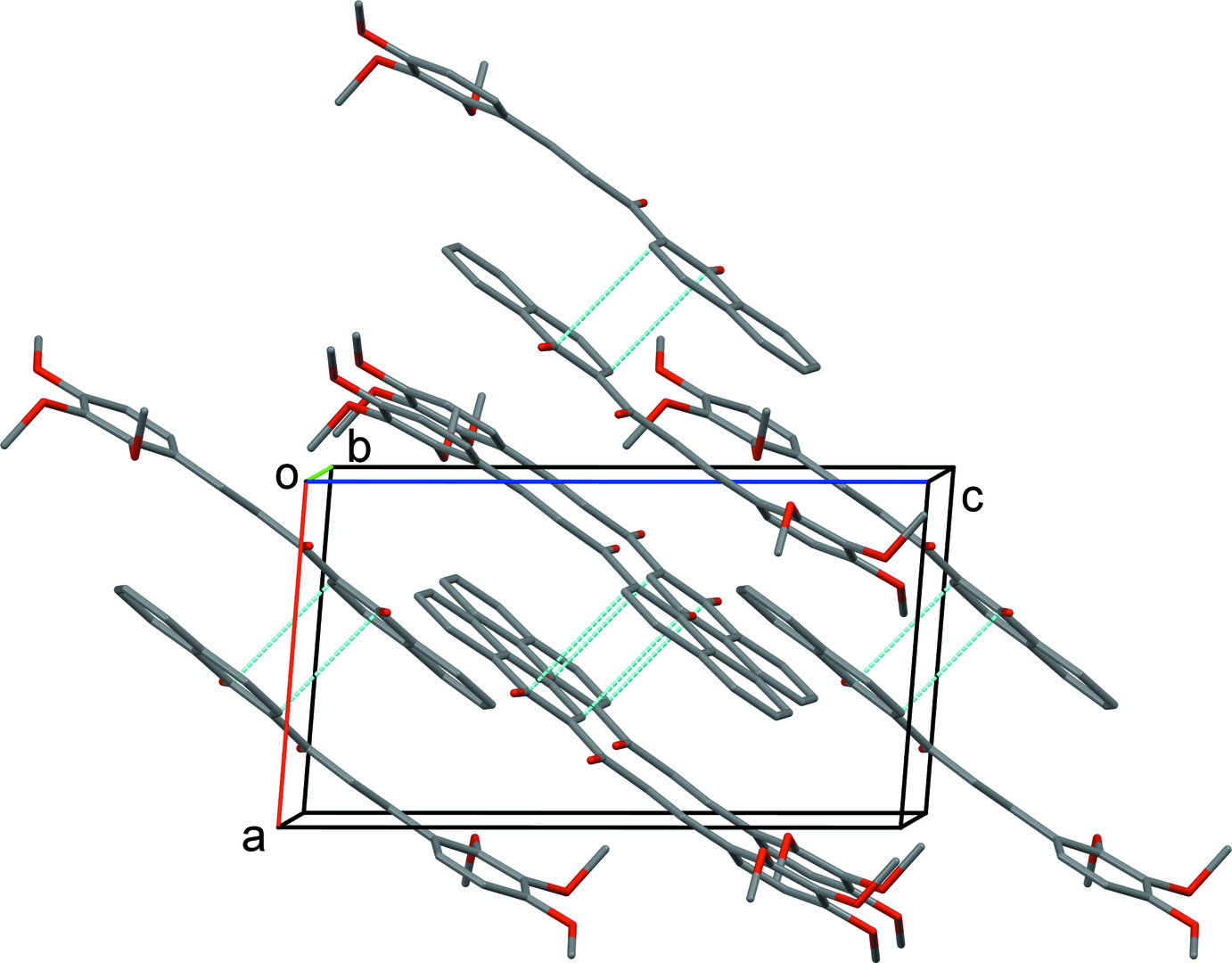

Supplement: Supplementary file 5 [file e-71-0o610-fig2.tif]
